# Long-term monitoring data from a naturally ventilated office building

**DOI:** 10.1038/s41597-019-0283-3

**Published:** 2019-11-26

**Authors:** Marcel Schweiker, Michael Kleber, Andreas Wagner

**Affiliations:** Building Science Group, Karlsruhe Institute of Technology, Englerstr. 7, 76131 Karlsruhe, Germany

**Keywords:** Energy modelling, Psychology and behaviour, Civil engineering

## Abstract

Data was collected in the field, from an office building located in Frankfurt, Germany, over the period of 4 years. The building was designed as a low-energy building and featured natural ventilation for individual control of air quality as well as buoyancy-driven night ventilation in combination with a central atrium as a passive cooling strategy. The monitored data include in total 116 data points related to outdoor and indoor environmental data, energy related data, and data related to occupancy and occupant behaviour. Data points representing a state were logged with the real timestamp of the event taking place, all other data points were recorded in 10 minute intervals. Data were collected in 17 cell offices with a size of ~20 m^2^, facing either east or west). Each office has one fixed and two operable windows, internal top light windows between office and corridor (to allow for night ventilation into the atrium) and sun protection elements (operated both manually and automatically). Each office is occupied by one or two persons.

## Abstract (summary)

**Table:** 

Measurement(s)	Ventilation • Occupancy • humidity • temperature of air • carbon dioxide
Technology Type(s)	Carbon Dioxide Monitor Device • sensor • Temperature Sensor Device • Electrically Open Reed Device
Factor Type(s)	indoor environment
Sample Characteristic - Organism	Homo sapiens
Sample Characteristic - Environment	office building
Sample Characteristic - Location	Germany

Machine-accessible metadata file describing the reported data: 10.6084/m9.figshare.9785606

## Background & Summary

The introduction of the European “Energy Performance of Buildings Directive” in 2001 gave a strong incentive to reduce excessive energy consumption through a holistic approach in terms of building design and integrated energy concepts. Within this context, the program SolarBau, introduced by the German Federal Ministry of Economic Affairs, funded ambitious demonstration projects within the non-residential building sector setting benchmarks in terms of low primary energy consumption. Buildings in Germany are benchmarked through their primary energy demand for heating, cooling, ventilation, lighting and domestic hot water (DHW). The monitored building described here had a projected value of 107 kWh/m²a.

Within this program, a strong focus was set on various passive cooling strategies in combination with a higher insulation standard than required by German regulations in the year of construction (see Table 1 for details). Daylight factors above standard at the workspaces were achieved by proper window design and light directing devices (venetian blinds with different blind positions and ceiling panels above the desks). At the same time, occupants’ interactions with windows and blinds – essential aspects in the context of passive cooling concepts – was addressed^1–3^ as well as their thermal comfort under these conditions. A two-year monitoring after commissioning of the building was compulsory for a proof of concept for all funded buildings.

**Table 1:** Building characteristics.

Type of building	Multi-storey office building
Dimension	17,402 m^2^ (8,585 m^2^ heated)
No. of Employee	~350 employees
Location	Frankfurt, Germany
Thermal characteristics	High energy standard of building envelope Walls: U-values 0.24 to 0.5 W/m^2^K) Windows: U-values 1.5 W/m^2^K, solar transmittance <40%, light transmittance 70%
Structural system	Reinforced concrete construction
Type of observed spaces	Office rooms
Year of construction	2002
No. of floors	2-level underground car park +4 office floors + 1 floor apartments on top
Window dimensions	Windows: Top lights
Windows, orientation	Mostly E and W
Window opening	All windows open inwards. No obstacles prevented window opening except those potentially added by occupants (e.g. plants or papers placed in front of window) Windows: Manual opening through window handle by occupants only, windows had hinges on one side and could be fully opened (rotated) to any degree up to 90° opening angle Top lights: automatic control + manual opening through switch next to office door by occupants, windows had hinges at the bottom and opened on top; any degree up to ° was possible at manual control; at automatic control for night-time ventilation the angle was predefined in order to balance pressure difference between floors and achieve almost the same volume flow for each floor and office.
Window control options	Automated 10 minutes flush ventilation before working hours through top lights in the façade and between office and corridor Afterwards: Top lights: Tilt (automatic + occupant driven mode), Windows: Tilt-and turn (occupant driven)
Shading devices	External sun protection (automatic + occupant driven mode) with different angle of blinds in the upper part to provide daylight guidance. Sun protection consists of light metal Venetian blinds with a slats width of 80 mm and a reflectance of 60%..
Predicted annual primary energy consumption	107 kWh/m²
Monitored annual primary energy consumption	100 kWh/m² in third year of monitoring

Influencing factors on the occupants’ behaviour with regard to the operation of windows and blinds are, among others, the indoor and outdoor environmental conditions such as temperatures, relative humidity levels, air quality levels, and lighting levels^4,5^. Due to their daily and seasonal variation, long-term monitoring data, i.e. at least a full year, is essential to capture their influence on occupants’ behavioural patterns.

The monitored building is located in Frankfurt am Main, Germany. Key characteristics of the building are presented in Table 1. An important design feature to enhance natural night ventilation is a large atrium with an extended “chimney” around which the offices are located. This enables a buoyancy-driven airflow from the windows through the offices themselves, into the traffic zones, and then up into the chimney where the air leaves the building. The airflows through the offices are levelled out by the opening angle of the top lights, located above the manually operable windows. Directly exposed concrete ceilings in the offices enable the activation of thermal mass as an essential part of passive cooling by night ventilation. Furthermore, the atrium increases the usage of natural lighting for the interior traffic zones.

Only the meeting rooms, the offices to the south behind the double-skin facade, offices with suspended ceilings and a number of special purpose areas are actively cooled, but are not part of this dataset.

**Fig. 1:**
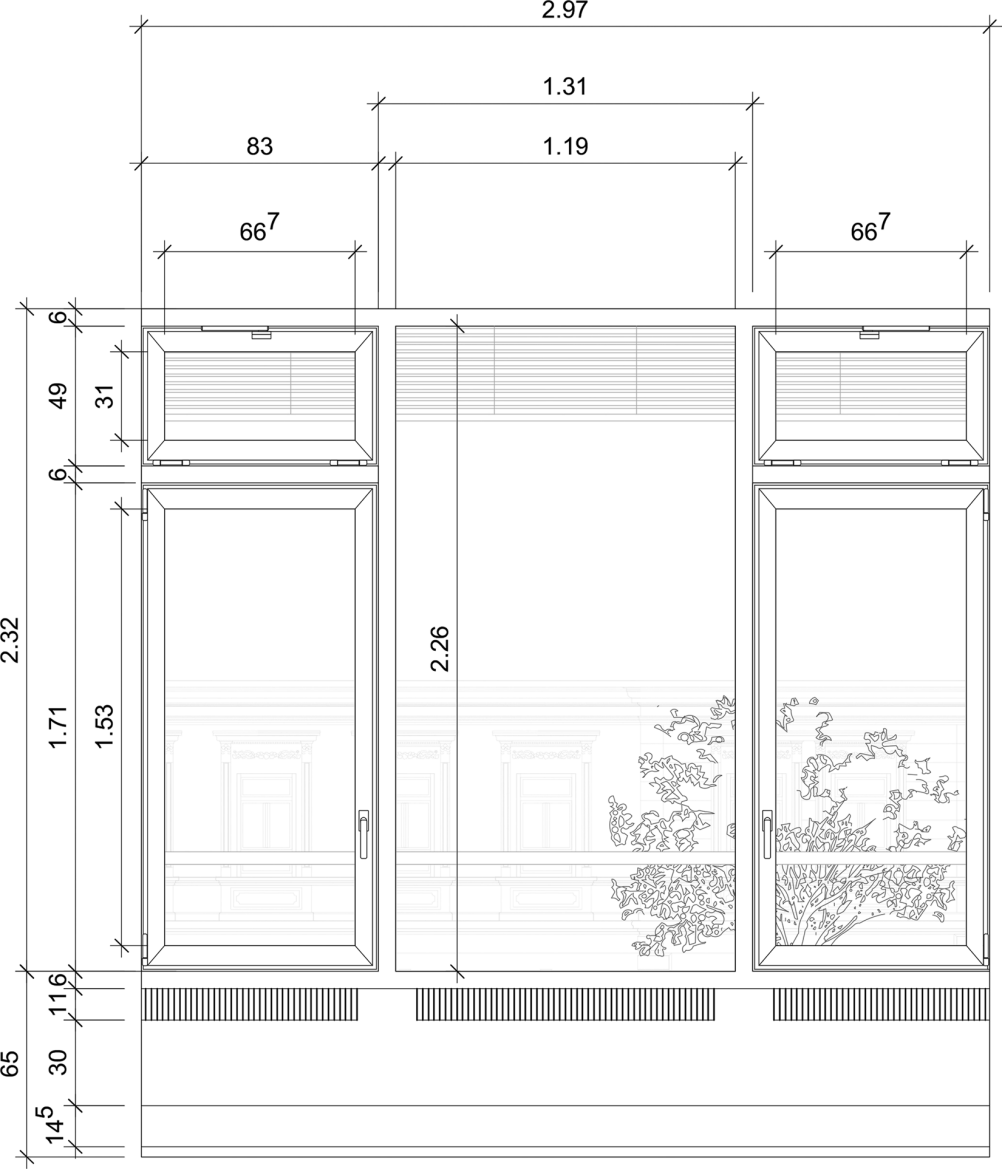
Window positions and dimensions of one office.

The occupant is able to open the windows manually. For operating the top–light windows (see Fig. 1) occupants have to use the control panel which is located beside the door (opposite to the façade). Through this panel, occupants can also control the exterior Venetian blinds and the artificial lighting of the office. Outdoor noise due to traffic was only present in the area of the south facing rooms, which are not part of this dataset. No other noise sources are known. Outdoor air quality was high, given outdoor CO_2_-levels (included in data) being mean 465 ppm ± 52 (standard deviation).

The dataset published consists of long-term data from January 2005 to December 2008, i.e. starting 2 years after the construction, when initial problems with controls where already solved. The data published has a 10-minute interval for continuous data and the event data with individual time stamps and consists of data from 17 offices.

The data has been analysed and used by several authors. A first analysis of the indoor environmental conditions and energy revealed that indoor air quality levels were high, that the primary energy consumption was at a low level as predicted, and that the monitoring was a useful measure towards an optimized operation^6,7^. Schakib-Ekbatan *et al.* questioned the fit between occupant behaviour and the building concept and found several occurrences of occupants’ window opening behaviour contradicting the natural ventilation concept^8^. An additional analysis of the data applied a data mining framework for identifying occupancy patterns and found four archetypal working profiles^9^.

## Methods

### Monitoring concept

In order to collect long-term data automatically and frequently, all sensors were connected to the building management system (BMS) of the building. Data were gathered in 10-minute intervals or as event data. Data was stored for one day locally and send at night as csv-files to the server of the researcher. Data was stored in a MySQL database.

A weather station was located on the top of the building at 2 m above the roof, i.e. around 30 m above street level. The weather station is providing data regarding the outdoor conditions for all offices, such as temperature or wind speed. However, the microclimate on the façades can differ, e.g. depending on the intensity and direction of wind. The precipitation meter was not heated. However, snowfall is seldom. There are no direct obstacles close-by affecting the wind speed or direction. Still, wind speed and direction might have been affected by the buildings in the neighbourhood.

All offices included in this data base have a size of ~20 m^2^ and are facing east or west (see also Table 2). Each office has one fixed and two operable windows with top light windows above, internal top light windows between office and corridor (to allow for night ventilation into the atrium) and external sun protection elements (operated both manually and automatically). One or two persons occupy each office.

**Table 2:** Orientation and variables available for each office.

	Room ID	Room air temperature	Occupancy	Window control	Top window control	Sun protection	Electricity demand lighting	Electricity demand plugs	CO_2−_ Concen- tration
East	E01	E01Tair	E01Occ	E01W	E01WT	E01SP			
	E02	E02Tair	E02Occ	E02W	E02WT	E02SP			
	E03	E03Tair	E03Occ	E03W	E03WT	E03SP			
	E04	E04Tair	E04Occ	E04W	E04WT	E04SP			E04CO2
	E05	E05Tair	E05Occ	E05W	E05WT	E05SP			
	E06	E06Tair	E06Occ	E06W	E06WT	E06SP	E06ElL	E06ElP	
	E07	E07Tair	E07Occ	E07W	E07WT	E07SP	E07ElL	E07ElP	E07CO2
	E08	E08Tair	E08Occ	E08W	E08WT	E08SP			
	E09	E09Tair	E09Occ	E09W	E09WT	E09SP			
	E10	E10Tair	E10Occ	E10W	E10WT	E10SP			
	E11	E11Tair	E11Occ	E11W	E11WT	E11SP			
West	W01	W01Tair	W01Occ	W01W	W01WT	W01SP	W01ElL	W01ElP	
	W02	W02Tair	W02Occ	W02W	W02WT	W02SP			
	W03	W03Tair	W03Occ	W03W	W03WT	W03SP			
	W04	W04Tair	W04Occ	W04W	W04WT	W04SP			
	W05	W05Tair	W05Occ	W05W	W05WT^a^	W05SP		W05ELP	W05CO2
	W06	W06Tair	W06Occ	W06W	W06WT	W06SP			

^a^Note that no data file is provided for this sensor, because no event was recorded over the monitoring period.

Presence of occupants was measured by an infrared sensor located in the middle of the ceiling panel, which is suspended from the ceiling above the work places.

Air temperature, relative humidity and CO_2_-level were measured inside each office close to the office door at 1.1 m height through a device attached to the walls separating the offices from the corridor.

Occupants can change the status of top-light windows, blinds, and lighting through a set of buttons close to the office door. Windows can be opened directly at the façade. Status of windows was measured through reed-contacts connected to the buildings’ BMS system. Position of the blinds was measured based on blinds’ motor run time.

The data points available in the database are presented in Table 3. These data can be grouped into outdoor environmental data, indoor environmental data, energy related data, and data related to occupancy and occupant behaviour.

**Table 3:** Variables, their categories and subcategories according to the ontology for building monitoring^13^, and intervals.

Categories of data	Subcategories of measured data	Variable	Interval	Sensor	
				Range	Accuracy
Inhabitants	Other	Presence (all rooms)	Event	—	—
	Other	Window state (open/closed)	Event	—	—
	Other	Top-light window state (open/closed)	Event		
	Other	State of sun protection (open/closed)	Event	—	—
Indoor conditions	Hygro-thermal	Air temperature	10 minutes	0–40 °C	±0.1 K
	Hygro-thermal	Relative humidity (all rooms)	10 minutes	0–100%	±1%
	Indoor Air Quality	CO_2_-level (3 rooms)	10 minutes	300–3500 ppm	±3%
External conditions	Hygro-thermal	Air temperature	10 minutes	−40–+ 45 °C	±0.1 K
	Hygro-thermal	Relative humidity	10 minutes	0–100%	±2%
	Visual	Illuminance (4 orientations + horizontal)	10 minutes	0–100,000 lx	±5%
	Solar radiation	Horizontal solar radiation	10 minutes	0–1300 W/m²	±2.5%
	Other	Precipitation (Amount and event)	10 minutes	—	—
	Other	Wind speed and direction	10 minutes	0–360° 0–20 m/s	—
Energy	Heating/cooling	Overall heat quantity pellet boiler and gas boiler	10 minutes		±1%
	Lighting	Lighting energy (3 meters for 5 rooms)	10 minutes		±0.5%
	Equipment	Plug loads separated by IT and other (3 rooms)	10 minutes		±0.5%
	Other	Total electricity use of building	10 minutes		±0.5%

## Data Records

All data records listed in this section are available from the project pages^10^ on Open Science Framework (OSF) and can be downloaded without an OSF account. We licensed the data under a CC0 1.0 Universal license.

### All datasets

File format: comma separated values file (.csv). Data is available as one file for each sensor including date and time column. All date formats are in the format day, month, year, i.e. dd.mm.yyyy. Devices in use are recorded with 1 or 100, those not in use with 0. This translates to open windows being in the data recorded as 1 and completely closed blinds with values 100.

### Code book

File format: comma separated values file (.csv).

## Technical Validation

Incoming datasets were analysed according to their completeness and validity. An error message was sent to the researchers in case these checks revealed problems. These analyses mainly targeted for checking availability of data and to filter implausible or missing values. Missing values in air temperature, relative humidity, and CO_2_ were marked by a value of “0” and filtered automatically. Implausible values, e.g. indoor air temperatures above 35 °C, were flagged by the monitoring software and manually inspected using the visualization tools of the monitoring software. The monitoring software used was MoniSoft^11^.

The air temperature sensors were checked and calibrated during commissioning by the facility management and later comparison through a high-quality comfort meter equipment in sample rooms showed good conformity. All other sensors had been calibrated by the manufacturer, but could not be calibrated again during operation.

## Usage Notes

By the general and open csv format the researcher is free to use whatever software s/he finds suitable for analysing or visualising the data. For comfort analysis the R-package comf is recommended^12^.

For further analyses, it needs to be considered, that top window and blind states were either changed through the BMS or manually by occupants. The algorithm of automatic controls of top windows and blinds is unknown. The authors assume that it will be possible to identify automated and manual controls by means of statistical analyses.

Window state and blind status changes were recorded by the BMS and are available with their original time stamp. Blind events are all changes, i.e. also changes of blind position e.g. between 20 and 80% closing.

Note that the official monitoring period by the original research team ended in October 2006. After that, data was still automatically received, but the status of sensors not checked anymore. Therefore, the number of sensors having failures and not providing data continuously increases, which needs to be considered when using data points after 2006.

## Data Availability

Custom code was used to validate the incoming data from the BMS for completeness and validity. The code had been very specific according to the system configuration and is not available anymore. Its value for future applications or future data usage would be very low because 90% of the code was written to check the syntactically correctness of the data. While the authors expected such syntactical correctness being granted for data exported from a BMS, the first month of monitoring (not included in the database) showed several problems with the structure of the data, which required many lines of custom code, very specific to the BMS in place and therefore not generalizable to any other application.
